# Impaired sleep quality mediates the relationship between internet gaming disorder and conduct problems among adolescents: a three-wave longitudinal study

**DOI:** 10.1186/s13034-025-00889-2

**Published:** 2025-03-21

**Authors:** Pu Peng, Jieyin Jin, Zhangming Chen, Silan Ren, Ying He, Jinguang Li, Aijun Liao, Linlin Zhao, Xu Shao, Shanshan Chen, Ruini He, Yudiao Liang, Youguo Tan, Xiaogang Chen, Jinsong Tang, Yanhui Liao

**Affiliations:** 1 Department of Psychiatry, Sir Run Run Shaw Hospital, Zhejiang University School of Medicine, Hangzhou, Zhejiang China; 2 Department of Psychiatry, Zigong Mental Health Center, Zigong, Sichuan China; 3 Department of Nursing, Sichuan Vocational College of Health and Rehabilitation, Zigong, Sichuan China; 4 Department of Psychiatry, National Center for Mental Disorders, National Clinical Research Center for Mental Disorders, The Second Xiangya Hospital of Central South University, Changsha, 410011 Hunan China; 5 East Qingchun Road, Hangzhou, Zhejiang P.R. China

**Keywords:** Internet gaming disorder, Adolescents, Conduct problems, Sleep quality, Longitudinal study

## Abstract

**Background:**

Research increasingly demonstrates a positive association between Internet Gaming Disorder (IGD) and conduct problems among adolescents. However, longitudinal data are limited, and the mediating mechanisms remain unclear. This study aimed to examine the predictive effect of IGD on conduct problems and explore the mediating role of impaired sleep quality.

**Method:**

A cohort of 20,137 Chinese seventh- and tenth-grade students was recruited and assessed at three time points: November 2020 (T1), 2021 (T2), and 2022 (T3). IGD, conduct problems, and impaired sleep quality were measured using the Internet Gaming Disorder Scale Short Form, the Strengths and Difficulties Questionnaire, and the Pittsburgh Sleep Quality Index, respectively. Mediation analyses were conducted to evaluate the direct and indirect effect of IGD on conduct problems, with subgroup analyses based on sex and developmental stage.

**Results:**

IGD was found to be an independent risk factor for conduct problems both cross-sectionally and longitudinally. Impaired sleep quality partially mediated the relationship between IGD and conduct problems, accounting for approximately 17.3% of the total effect. Subgroup analyses revealed that the mediation effect of impaired sleep quality was more pronounced in early adolescents and varied by sex, with a stronger total and direct effect in boys.

**Conclusions:**

These findings highlight the need for comprehensive interventions targeting both IGD and impaired sleep quality, tailored to specific sexes and developmental stages, to effectively reduce conduct problems.

**Supplementary Information:**

The online version contains supplementary material available at 10.1186/s13034-025-00889-2.

## Introduction

Conduct problems, encompassing behaviors such as aggression, rule-breaking, and antisocial activities, present a significant public health challenge among adolescents [1]. Affecting approximately 5–10% of adolescents globally, these behaviors can lead to both immediate and long-term consequences [2–4]. Beyond behavioral issues, adolescents with conduct problems often experience academic difficulties, strained social relationships, and impaired mental health [5]. Over time, these early behavioral challenges can develop into adverse outcomes in adulthood, including criminal behavior, substance abuse, and chronic mental health disorders [6–8]. Additionally, recent studies have highlighted the substantial economic burden of adolescent conduct problems, which includes increased healthcare costs, reduced productivity, and expenses related to the criminal justice system [9, 10]. Hence, it is crucial to identify and address the risk factors contributing to conduct problems.

### Association between internet gaming disorder and conduct problems

Internet Gaming Disorder (IGD) has emerged as a growing concern among adolescents [11]. IGD is characterized by excessive and compulsive gaming behaviors that result in significant impairment or distress. It is associated with various negative outcomes, including social isolation, impaired sleep quality, depression, anxiety, academic failure, and suicidality [12–14]. Emerging research has identified a positive relationship between IGD and conduct problems [15–17]. However, longitudinal studies examining this association are limited and have produced mixed results [18–21]. While some research suggests that IGD can predict future conduct problems [19], other studies have not found a significant long-term relationship [18]. These inconsistent findings highlight the need for large-scale longitudinal studies to clarify the temporal relationship between IGD and conduct problems.

### Impaired sleep quality as potential mediator between IGD and conduct problems

A key gap in the current literature is the limited understanding of the mechanisms linking IGD to conduct problems. Most existing studies have focused on the direct relationship without exploring potential mediating factors. Impaired sleep quality is a promising candidate for mediation, which is a common consequence of behavioral addictions including IGD, social media addiction, and smartphone addiction [22, 23]. Longitudinal research indicates that IGD often leads to sleep problems, such as reduced sleep duration and poorer sleep quality [12]. These sleep issues can, in turn, result in increased irritability, impaired emotional regulation, and heightened aggression, all of which contribute to conduct problems [24, 25]. Preliminary support for this mediation comes from cross-sectional studies. For example, a large-scale American study found that increased screen time was associated with aggressive and rule-breaking behaviors, with shorter sleep duration mediating this relationship [26]. Another study reported that insomnia fully mediated the relationship between IGD and aggression in youths aged 18 to 24 years [27]. However, these studies are limited by their cross-sectional design, which does not allow for establishing temporal order. To date, no longitudinal research has rigorously tested whether impaired sleep quality mediates the relationship between IGD and conduct problems.

### Investigating differences across sex and developmental stages in the interrelationship between IGD, conduct problems, and impaired sleep quality

Both IGD and conduct problems are more prevalent among boys, and recent evidence suggests that their relationship may differ by sex. For instance, one study found a reciprocal relationship between IGD and aggression in boys but not in girls [21], highlighting the importance of considering sex differences in future research. Additionally, developmental stage may moderate the association between IGD and mental health outcomes. Research indicates that the impact of IGD on mental health may vary between early and late adolescence [28–30]. A recent study showed that the relationship between IGD, depression, and anxiety differed between middle (junior high) and late (senior high) adolescents [28]. Furthermore, a meta-analysis reported that the link between IGD and aggression is stronger in younger adolescents compared to older youths, such as college students [17].These findings emphasize the need to examine how sex and developmental stage influence the relationship between IGD, conduct problems, and impaired sleep quality.

### The current study

To bridge these gaps, we conducted the present three-wave longitudinal study among a substantial sample of Chinese adolescents (*N* = 20137), aiming to test the following hypotheses: (1) Is IGD an independent risk factor for conduct problems among adolescents? (2) Does impaired sleep quality mediate the relationship between IGD and conduct problems? (3) Is there a difference in the association between IGD, conduct problems, and impaired sleep quality across sex and developmental stages? These subgroup analyses were explanatory in nature.

## Method

### Study procedure and participants

This longitudinal, school-based study was conducted in Zigong City, located in southwestern China. Detailed information on the sampling process, data collection methods, and quality assurance procedures has been previously published [31–33]. In November 2020 (Wave 1, T1), we recruited a total of 13,361 seventh-grade and 6,776 tenth-grade students from 76 schools using a two-step cluster sampling approach. Initially, all eligible middle school principals in the selected area were invited to participate. Subsequently, students from each participating school were enrolled. These participants were followed up in November 2021 (Wave 2, T2) and November 2022 (Wave 3, T3). Data were collected through electronic questionnaires, which students completed in their schools’ computer centers during regular school hours.

The study was conducted in accordance with the Declaration of Helsinki and received ethical approval from the Zigong Mental Health Center (Approval No. 2020-8-01). Written informed consent was obtained from all participants, and for those under 18, consent was also obtained from a parent or legal guardian.

### Measurements

#### Predictor at T1

Internet Gaming Disorder (IGD) was assessed at baseline using the nine-item Internet Gaming Disorder Scale - Short Form (IGDS9-SF) [34]. Participants responded to each item on a five-point Likert scale (1 = never to 5 = always), indicating the frequency of gaming-related behaviors over the past year. Higher scores reflect greater severity of IGD. Based on prior research, participants were categorized into three groups: adolescents without problematic gaming (< 21), risky gamers (21–31), and gamers with IGD (32 or above) [35, 36]. This classification captures varying levels of IGD severity. The IGDS9-SF has demonstrated strong reliability and validity in Chinese populations [37].

#### Outcome at T1, T2 and T3

Conduct problems were assessed using the conduct problems subscale from the Strengths and Difficulties Questionnaire (SDQ), which is widely used to evaluate internalizing (peer problems, emotional problems) and externalizing (conduct problems, hyperactivity/inattention) issues in children and adolescents [38]. The conduct problems subscale (SDQ-CP) comprises five items that measure symptoms of aggression, rule-breaking, and antisocial tendencies, each scored on a three-point scale (0 = not true, 1 = somewhat true, 2 = certainly true). Higher scores indicate more severe conduct problems. A cutoff score of 5 was used to identify clinically significant conduct problems, in line with previous validation studies in Chinese adolescent populations [39].

#### Mediators at T1 and T2

Impaired sleep quality was measured at T1 and T2 using the Pittsburgh Sleep Quality Index (PSQI), a widely recognized instrument for assessing sleep-related problems over the past month [40]. The PSQI includes 19 self-reported items that generate seven component scores: subjective sleep quality, sleep latency, sleep duration, habitual sleep efficiency, sleep disturbance, use of sleep medication, and daytime dysfunction. Each component is scored from 0 to 3, with higher scores indicating poorer sleep quality. A global PSQI score is obtained by summing the component scores, and a score of ≥ 6 signifies impaired sleep quality [41]. The total scores of PSQI were used in all subsequent analysis. The PSQI has demonstrated strong reliability and validity in the Chinese population [41].

#### Covariates

Baseline demographic variables included gender, age, place of residence, grade level, and family-related factors such as only-child status, left-behind status, and family structure (single-parent or blended family). Participants also reported on smoking and alcohol consumption behaviors. Additional mental health covariates included the peer problems, emotional symptoms, and hyperactivity/inattention subscales of the SDQ [39]. Participants scoring ≥ 6 on peer problems, ≥ 7 on emotional symptoms, and ≥ 7 on hyperactivity/inattention were classified as experiencing clinically significant distress in each respective domain. These covariates were found to be closely associated with conduct problems in prior research [42–46].

### Statistical analysis

Descriptive statistics were calculated for all variables, with means and standard deviations presented for continuous variables and frequencies and percentages for categorical variables. Baseline characteristics were compared between adolescents with and without conduct problems using Chi-square tests for categorical variables and Student’s t-tests for continuous variables.

Logistic regression models were then employed to examine both cross-sectional and longitudinal associations between baseline IGD and conduct problems across the three time points. These models controlled for baseline demographic factors and mental health issues. In the longitudinal analysis, we additionally adjusted for baseline conduct problems, which ensured that the observed association between IGD, impaired sleep quality, and later conduct problems reflected their unique contribution, rather than pre-existing behavioral tendencies.

Subsequently, a mediation analysis was conducted with T1 IGDS9-SF scores as the predictor, T2 PSQI total scores as the mediator, and T3 SDQ-CP scores as the outcome for participants with at least one follow-up wave. Missing data were handled using full information maximum likelihood, which estimated parameters using all available data and enhanced statistical efficiency without the need for data imputation [47]. Path coefficients were defined as follows: “a” for the effect of T1 IGD on T2 impaired sleep quality, “b” for the effect of T2 impaired sleep quality on T3 conduct problems, and “c” for the direct effect of T1 IGD on T3 conduct problems. The indirect effect was calculated as the product of paths (a * b). A bootstrap procedure with 5,000 iterations was used to generate 95% confidence intervals for both direct and indirect effects, with effects considered significant if the confidence intervals did not include zero. Both unadjusted and adjusted models were tested. As an additional robustness check, we reran the mediation analysis using only participants with complete data across all waves.

Finally, multiple-group analyses were performed to explore differences by gender (boys vs. girls) and developmental stage (seventh-grade as early adolescents vs. tenth-grade as late adolescents). Chi-square difference tests were used to compare the fit of an unconstrained model against models with paths constrained to be equal across the two groups.

All descriptive analyses, group comparisons, and logistic regressions were conducted using R (version 4.20). Mediation analyses were performed using Mplus (version 8.3). Statistical significance was set at *p* < 0.05 for all two-tailed tests.

## Results

### Sample characteristics

Table S1 presents the baseline characteristics of participants across all three waves. Initially, 20,137 seventh and tenth-grade students were enrolled in the study. At the first follow-up (T2), 15,061 students participated (response rate: 75%), and at the second follow-up (T3), 14,706 students completed the survey (response rate: 73%). Approximately 85% of the adolescents participated in at least one follow-up (*n* = 16,982), and 63% (*n* = 12,785) participated in all three waves. There were no significant differences in the main study variables—IGD status, conduct problems, and sleep quality—between participants who remained in the study and those who dropped out. However, dropout was slightly more likely among girls, younger students, only children, those with parents of higher education, and seventh-grade students, although these differences were minimal.

At baseline, the prevalence of conduct problems was 9.0% (Table 1). Adolescents with conduct problems were significantly younger (mean age = 13.24 ± 1.36 years) compared to those without (mean age = 13.42 ± 1.46 years). A higher proportion of boys (53.0% vs. 49.1%) and seventh-grade students (73.7% vs. 65.6%) exhibited conduct problems compared to their counterparts without conduct problems (*p* < 0.05). Additionally, conduct problems were more prevalent among only children (28.0% vs. 22.0%), those from single-parent or remarried families (26.5% vs. 20.1%), and those engaged in substance use (alcohol: 31.6% vs. 15.4%; smoking: 18.5% vs. 5.8%, *p* < 0.001). Psychologically, adolescents with conduct problems reported more severe impaired sleep quality (PSQI: 6.0 ± 3.8 vs. 3.8 ± 3.0) and higher IGD scores (IGDS9-SF: 19.8 ± 8.7 vs. 14.5 ± 6.0, *p* < 0.001). Furthermore, the prevalence of emotional problems, peer problems, and hyperactivity/inattention was significantly higher among those with conduct problems.

**Table 1:** Sample characteristics by baseline conduct problems

Characteristic	Overall, *N* = 20,137^a^	Without conduct problems, *N* = 18,328^a^	With conduct problems, *N* = 1,809^a^	*p*-value^b^
Age	13.40 ± 1.45	13.42 ± 1.46	13.24 ± 1.36	< 0.001
Gender				0.002
Boys	9,952 (49.4%)	8994 (49.1%)	958 (53.0%)	
Girls	10,185 (50.6%)	9334 (50.9%)	851 (47.0%)	
Grade				< 0.001
7	13,361 (66.4%)	12,027 (65.6%)	1,334 (73.7%)	
10	6776 (33.6%)	6301 (34.4%)	475 (26.3%)	
Residence				0.445
Urban	7237 (35.9%)	6572 (35.9%)	665 (36.8%)	
Country	12,900 (64.1%)	11,756 (64.1%)	1144 (63.2%)	
Single child				< 0.001
No	15,606 (77.5%)	14,304 (78.0%)	1302 (72.0%)	
Yes	4531 (22.5%)	4024 (22.0%)	507 (28.0%)	
Left-behind child				0.074
No	13,383 (66.5%)	12,215 (66.6%)	1168 (64.6%)	
Yes	6754 (33.5%)	6113 (33.4%)	641 (35.4%)	
Father education level				0.248
Below high school	15,453 (76.7%)	14,045 (76.6%)	1408 (77.8%)	
High school or above	4684 (23.3%)	4283 (23.4%)	401 (22.2%)	
Mother education level				0.724
Below high school	16,032 (79.6%)	14,586 (79.6%)	1,446 (79.9%)	
High school or above	4105 (20.4%)	3742 (20.4%)	363 (20.1%)	
Family type				< 0.001
Nuclear family	15,977 (79.3%)	14,648 (79.9%)	1329 (73.5%)	
Single parent or remarried	4160 (20.7%)	3680 (20.1%)	480 (26.5%)	
Alcohol use				< 0.001
Without	16,742 (83.1%)	15,505 (84.6%)	1237 (68.4%)	
With	3395 (16.9%)	2823 (15.4%)	572 (31.6%)	
Smoking use				< 0.001
Without	18,743 (93.1%)	17,268 (94.2%)	1475 (81.5%)	
With	1394 (6.9%)	1060 (5.8%)	334 (18.5%)	
PSQI scores	4.0 ± 3.2	3.8 ± 3.0	6.0 ± 3.8	< 0.001
Impaired sleep quality				< 0.001
Without	14,461 (71.8%)	13,578 (74.1%)	883 (48.8%)	
With	5676 (28.2%)	4750 (25.9%)	926 (51.2%)	
IGDS9-SF scores	15.0 ± 6.5	14.5 ± 6.0	19.8 ± 8.7	< 0.001
IGD status				< 0.001
Adolescents without problematic gaming	16,602 (82.4%)	15,556 (84.9%)	1046 (57.8%)	
Risky gamer	3019 (15.0%)	2435 (13.3%)	584 (32.3%)	
Gamer with IGD	516 (2.6%)	337 (1.8%)	179 (9.9%)	
Hyperactivity/inattention symptoms				< 0.001
Without	18,155 (90.2%)	1268 (70.1%)	16,887 (92.1%)	
With	1982 (9.8%)	541 (29.9%)	1441 (7.9%)	
Emotional problems				< 0.001
Without	18,088 (89.8%)	16,856 (92.0%)	1232 (68.1%)	
With	2049 (10.2%)	1472 (8.0%)	577 (31.9%)	
Peer problems				< 0.001
Without	18,485 (91.8%)	17,011 (92.8%)	1474 (81.5%)	
With	1652 (8.2%)	1317 (7.2%)	335 (18.5%)	

^a^Mean ± SD; n (%)

^b^Welch Two Sample t-test; Pearson’s Chi-squared test

### Cross-sectional and longitudinal association between IGD and conduct problems

**Fig. 1:**
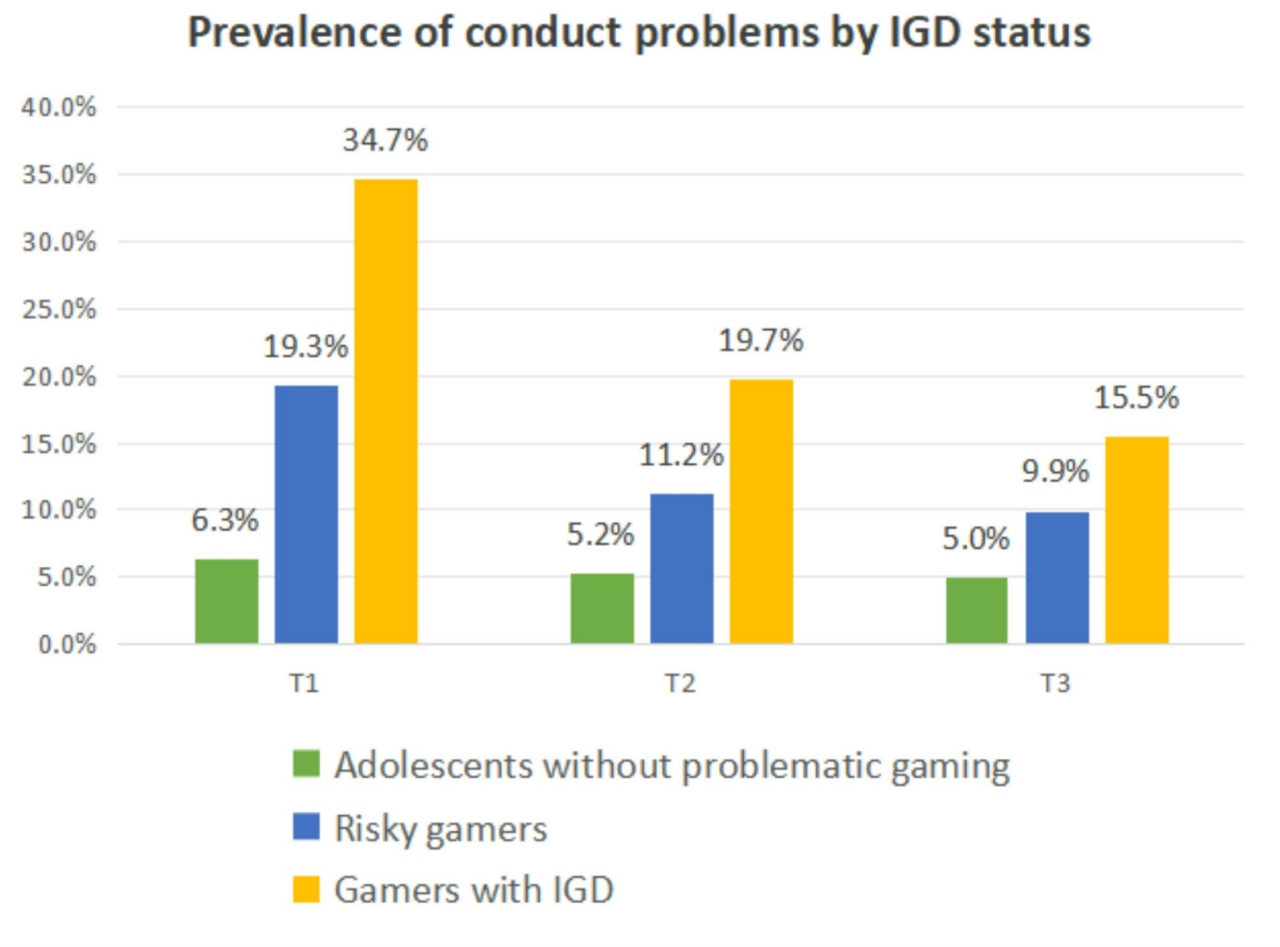
The prevalence of conduct problems by baseline IGD status

The prevalence of conduct problems decreased over time, from 9.0% at T1 to 6.5% at T2 and 6.0% at T3. As illustrated in Fig. 1, both risky gamers and gamers with IGD consistently reported higher prevalence rates of conduct problems compared to healthy gamers across all three time points. Specifically, at baseline, 34.7% of gamers with IGD and 19.3% of risky gamers exhibited conduct problems, compared to 6.3% of healthy gamers. Similar trends were observed at T2 and T3.

Logistic regression analyses revealed that both cross-sectional and longitudinal associations between IGD and conduct problems remained significant after adjusting for baseline covariates (Table 2). Compared to adolescents without problematic gaming, risky gamers had increased odds of conduct problems at T1 (adjusted odds ratio [OR] = 1.41, 95% CI = 1.13–1.77, *p* = 0.003), T2 (adjusted OR = 1.33, 95% CI = 1.12–1.58, *p* = 0.001), and T3 (adjusted OR = 1.32, 95% CI = 1.10–1.58, *p* = 0.003). IGD gamers exhibited even higher odds at T1 (adjusted OR = 3.01, 95% CI = 2.41–3.76, *p* < 0.001), T2 (adjusted OR = 1.63, 95% CI = 1.20–2.22, *p* = 0.002), and T3 (adjusted OR = 1.52, 95% CI = 1.09–2.11, *p* = 0.013).

**Table 2:** Association of IGD and impaired sleep quality with conduct problems

Characteristics	Conduct problems at T1		Conduct problems at T2		Conduct problems at T3	
	Unadjusted model	Adjusted model^a^	Unadjusted model	Adjusted model^b^	Unadjusted model	Adjusted model^b^
IGD status						
Adolescents without problematic gaming	—	—	—	—	—	—
Risky gamer	2.22 (1.81,2.71) ***	1.41 (1.13,1.77) **	2.29 (1.97,2.67) ***	1.33 (1.12,1.58) **	2.10 (1.78,2.47) ***	1.32 (1.10,1.58) **
Gamer with IGD	7.90 (6.52,9.57) ***	3.01 (2.41,3.76) ***	4.43 (3.37,5.82) ***	1.63 (1.20,2.22) **	3.48 (2.58,4.69) ***	1.52 (1.09,2.11) *
Impaired sleep quality	3.00 (2.72,3.31) ***	1.40 (1.24,1.58) ***	2.30 (2.02,2.62) ***	1.49 (1.28,1.74) ***	2.06 (1.80,2.37) ***	1.48 (1.26,1.74) ***

*: *p* < 0.05; **: *p* < 0.01;***:*p* < 0.001

^a^ Adjusted for baseline demographics and mental health covariates

^b^ Adjusted for baseline demographics, mental health covariates, and conduct problems

Additionally, baseline impaired sleep quality was significantly associated with increased odds of conduct problems at all three time points: T1 (adjusted OR = 1.40, 95% CI = 1.24–1.58, *p* < 0.001), T2 (adjusted OR = 1.49, 95% CI = 1.28–1.74, *p* < 0.001), and T3 (adjusted OR = 1.48, 95% CI = 1.26–1.74, *p* < 0.001).

### The mediating role of impaired sleep quality between IGD and conduct problems

**Fig. 2:**
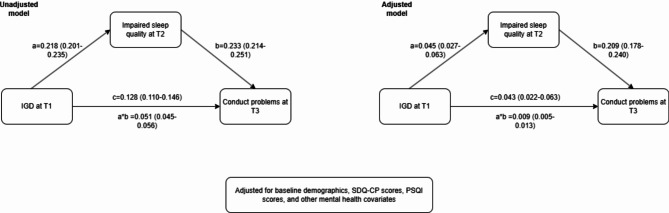
The unadjusted and adjusted mediation model in all samples

The mediation analysis revealed both direct and indirect effects of IGD on conduct problems (Fig. 2). In the unadjusted model, IGD at T1 was significantly associated with impaired sleep quality at T2 (a = 0.218, 95% CI = 0.201–0.235), which in turn were associated with increased conduct problems at T3 (b = 0.233, 95% CI = 0.214–0.251). The direct effect of IGD on conduct problems (c) was 0.128 (95% CI = 0.110–0.146), and the indirect effect (a × b) was 0.051 (95% CI = 0.045–0.056). This indicates that impaired sleep quality mediated 28.5% of the total effect in the unadjusted model. After adjusting for baseline covariates, all paths remained significant. The direct effect was reduced to 0.043 (95% CI = 0.022–0.063), and the indirect effect decreased to 0.009 (95% CI = 0.005–0.013), accounting for 17.3% of the total effect.

Sensitivity analysis using data from participants with complete data across all waves produced consistent results. In the fully adjusted model, the direct effect of IGD on conduct problems was 0.041 (95% CI = 0.020–0.062), and the indirect effect via impaired sleep quality was also significant (β = 0.010, 95% CI = 0.006–0.014). These findings further support the reliability of our mediation model.

### Subgroup analysis based on sex and developmental stages

**Fig. 3:**
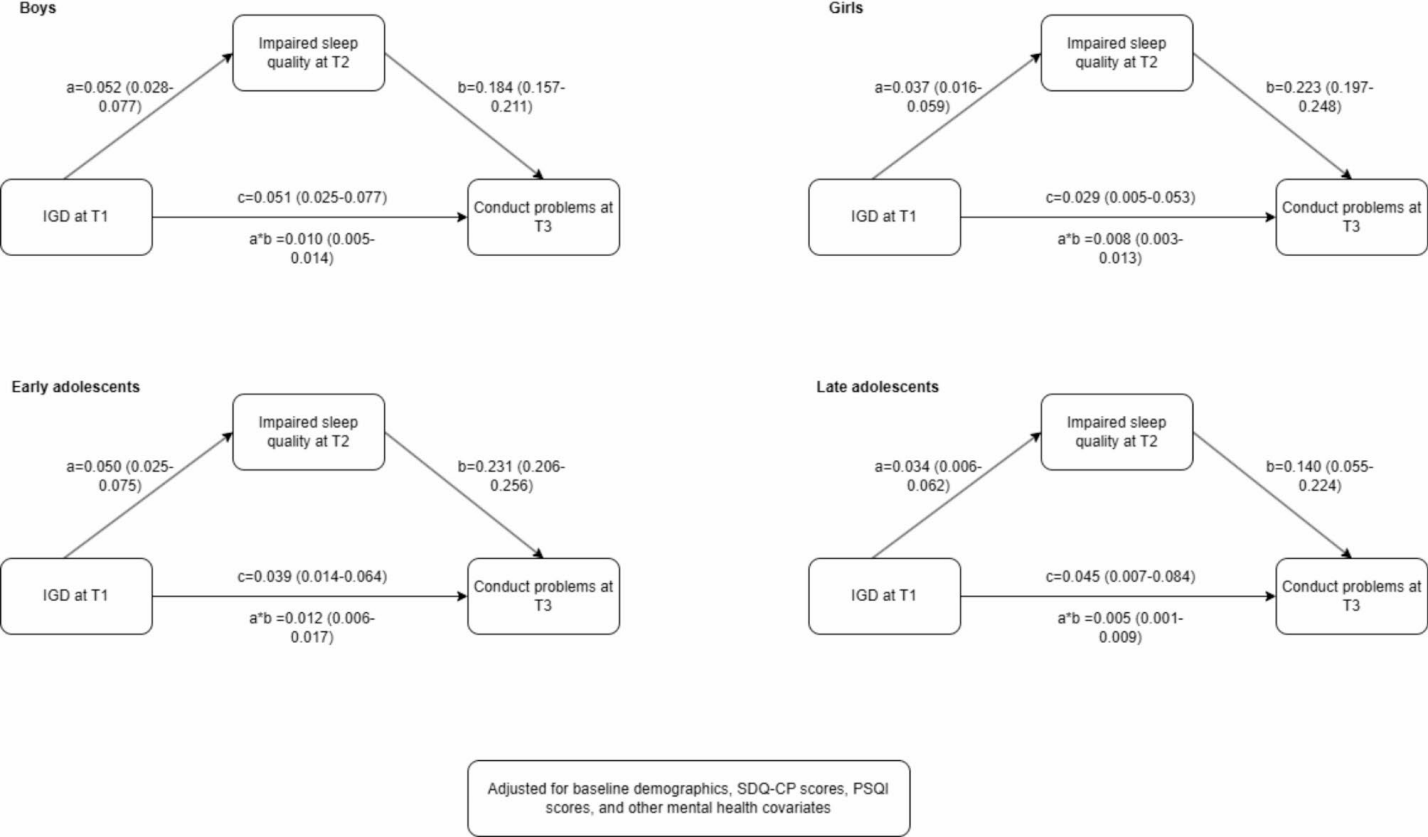
The mediation model across sex (boys and girls) and developmental stages

Figure 3 displayed the results of subgroup analysis. Multiple-group analyses revealed significant differences in the mediation model between boys and girls (Δχ^2^ = 101.266, Δdf = 33, *p* < 0.001). While in both girls and boys, the direct and indirect effect of IGD on conduct problems were significant, both the total effect and direct effect was greater in boys (total effect = 0.061, direct effect = 0.051) compared to girls (total effect = 0.037, direct effect = 0.029). The mediation proportion of impaired sleep quality was slightly higher among girls (21.6%) compared to boys (16.4%).

There were also significant differences in the mediation model between early and late adolescents (Δχ^2^ = 137.718, Δdf = 33, *p* < 0.001). Both early (seventh-grade) and late (tenth-grade) adolescents showed significant direct and indirect effects of IGD on conduct problems, with comparable total effects. However, the proportion of the mediation effect via impaired sleep quality was higher among early adolescents (23.5%) compared to late adolescents (10%). This indicates that impaired sleep quality played a more substantial mediating role in the IGD-conduct problems relationship during early adolescence.

## Discussion

This three-wave cohort study examined the prospective association between IGD and conduct problems and explored the mediating effect of impaired sleep quality among Chinese adolescents. The key findings of our study are threefold: (1) IGD is an independent risk factor for conduct problems; (2) Impaired sleep quality partially mediate the relationship between IGD and conduct problems, accounting for approximately one-fifth of the total effect; and (3) The IGD-sleep-conduct problems relationship varies significantly across sex and developmental stages.

### Cross-sectional and longitudinal association between IGD and conduct problems

At baseline, the prevalence of conduct problems and IGD was 9.0% and 2.6%, respectively, consistent with recent large-scale epidemiological data on Chinese adolescents [3, 48, 49]. While most prior research on the association between IGD and conduct problems has been conducted in Western populations [15, 16], our findings replicate these associations within a substantial Chinese sample. We observed a strong cross-sectional and longitudinal relationship between IGD and conduct problems. Notably, even adolescents classified as risky gamers, who did not meet the criteria for IGD, exhibited a higher risk of concurrent and future conduct problems. These results suggest that even subclinical levels of problematic gaming are linked to increased behavioral issues. This cross-cultural consistency underscores the universal nature of the IGD-conduct problems relationship and highlights the importance of screening for conduct problems among adolescents engaged in problematic gaming behaviors, including both risky gamers and those with IGD.

Several explanations may account for the substantial association between IGD and conduct problems. First, excessive gaming may displace time spent on social activities [50, 51], leading to reduced social skills and increased interpersonal conflicts, such as family disputes and peer bullying [52], which can contribute to conduct problems. Second, adolescents with IGD often struggle with difficulties in emotional regulation and expression and possess inadequate coping strategies [53–55]. Reliance on gaming for emotional regulation may hinder the development of healthier coping mechanisms, exacerbating behavioral issues [56]. Finally, from a neurobiological perspective, IGD is associated with alterations in brain regions involved in executive functioning [57–59], such as the prefrontal cortex, which is critical for impulse control and decision-making. Structural and functional changes in these areas may impair adolescents’ ability to regulate behaviors and adhere to social norms, thereby fostering conduct problems.

### The indirect effect of impaired sleep quality

Our study found that impaired sleep quality is an independent predictor of conduct problems, consistent with prior research [60]. Poor sleep disrupts emotional regulation, resulting in heightened irritability, impulsivity, and aggression [24, 25].It also impairs executive functions, such as impulse control and decision-making [61], which increases the likelihood of risk-taking and rule-breaking behaviors. These effects may be underpinned by biological mechanisms, including dysregulation of the hypothalamic-pituitary-adrenal axis, alterations in the brain’s cholinergic system, and structural changes in key brain regions [62–64]. Together, these pathways highlight the critical role of sleep disturbances in the development and exacerbation of conduct problems.

Consistent with our hypothesis, impaired sleep quality significantly mediated the relationship between IGD and conduct problems, accounting for about one-fifth of the total effect after adjusting for covariates. This finding contributes to the growing body of literature recognizing impaired sleep quality as a crucial pathway linking IGD to a series of adverse mental health outcomes, including depression, suicidality, psychotic-like experiences, and impaired well-being [65–69]. Collectively, these results highlight the importance of incorporating sleep assessments and interventions into strategies aimed at mitigating the behavioral and psychological impacts of IGD. Comprehensive intervention programs that target both gaming behaviors and sleep health could more effectively support the well-being of affected adolescents.

### The difference across sex and developmental stages

Our study revealed significant sex differences in the IGD-sleep-conduct problems relationship. Specifically, both the direct and total effects of IGD on conduct problems were more pronounced among boys, indicating that IGD may be a stronger predictor of conduct problems in this group. These findings partially align with those of Kim et al. [21], who observed a longitudinal association between IGD and aggression in Korean adolescent boys but not in girls. One possible explanation for these sex differences is the type of games played; boys may engage more frequently in aggressive and violent gaming, which could potentially lead to higher conduct problems [70]. Additionally, previous research on IGD and depression has produced similar results, finding a stronger effect of IGD on later depression among boy [71, 72], though the results were not entirely consistent [29, 73]. Collectively, these findings might suggest that boys may be more vulnerable to IGD-related mental distress. Future studies should explore the underlying biological and psychosocial mechanisms contributing to these sex differences.

Regarding developmental stages, the mediation effect of impaired sleep quality was more pronounced among early adolescents (seventh-grade students) compared to late adolescents (tenth-grade students). While the total effects of IGD on conduct problems were comparable across both groups, impaired sleep quality accounted for a larger proportion of this relationship in early adolescents. This suggests that younger adolescents may be more vulnerable to the negative impacts of IGD on sleep, which in turn affects their behavioral outcomes. Early adolescence is a critical period for developing sleep patterns and behavioral regulation; thus, interventions targeting sleep hygiene may be particularly beneficial for this age group.

### Clinical implication of the present study

Our study has several public health and clinical implications. The robust cross-sectional and longitudinal associations between IGD and conduct problems underscore the necessity for early identification and targeted interventions for adolescents engaged in problematic gaming. Recognizing IGD as a substantial risk factor for conduct problems emphasizes the importance of screening for both conditions in educational and clinical settings. Comprehensive screening programs that include assessments for IGD, conduct problems, and sleep quality can facilitate timely and effective interventions. The mediating effect of impaired sleep quality between IGD and conduct problems suggests that targeting sleep problems simultaneously with gaming behaviors may be an effective strategy for reducing gaming-related conduct problems. For example, interventions could integrate cognitive-behavioral therapy for insomnia (CBT-I) to improve sleep quality alongside CBT for IGD to address compulsive gaming. Additionally, psychoeducational programs could teach adolescents and parents about the importance of sleep hygiene and healthy screen time habits. School-based initiatives, such as sleep awareness campaigns and structured gaming schedules, could further reinforce these efforts. Future research is needed to validate the efficacy of such integrated approaches. Tailored intervention strategies are warranted based on subgroup analyses: girls and early adolescents may benefit more from interventions targeting impaired sleep quality, whereas boys and late adolescents might require more direct interventions addressing IGD itself.

### Strength and limitations

The present study boasts several strengths, including a large sample size and a robust three-wave longitudinal design, which enhance the reliability and generalizability of the findings. However, several limitations must be acknowledged. First, the reliance on self-reported measures may introduce response biases, such as social desirability or recall bias, potentially affecting data accuracy. Second, the study’s focus on Chinese adolescents may limit the generalizability of the findings to other cultural or geographical populations. Third, although impaired sleep quality was examined as a mediator, other potential mediators, such as emotional regulation, family dysfunction, and cyberbullying, were not explored and may also play critical roles in the IGD-conduct problems relationship. Additionally, while our three-wave longitudinal design supports temporal inferences, the observational nature of the study limits causal conclusions, and reverse causation remains a possibility. Future research should address these limitations by incorporating objective measures, diverse populations, and additional mediating factors to further clarify the mechanisms linking IGD to conduct problems.

## Conclusion

Internet gaming disorder is an independent risk factor for conduct problems among adolescents, with impaired sleep quality serving as a partial mediator in this relationship. Significant differences emerged across sex and developmental stages in the IGD-sleep-conduct problems association. Addressing both IGD and associated impaired sleep quality holds promise for reducing conduct problems. Additionally, interventions tailored to specific sexes and developmental stages may be warranted to enhance their effectiveness.

## Electronic supplementary material

Below is the link to the electronic supplementary material.

Supplementary Material 1

## Data Availability

The data was available on request from the corresponding author.

## Abbreviations

IGD: Internet gaming disorder
SDQ: Strengths and Difficulties Questionnaire
SDQ-CP: the conduct problem subscale of Strengths and Difficulties Questionnaire
PSQI: Pittsburgh Sleep Quality Index
