# Supplementary material for: Impaired sleep quality mediates the relationship between internet gaming disorder and conduct problems among adolescents: a three-wave longitudinal study
Source: Child Adolesc Psychiatry Ment Health. 2025 Mar 21;19:26. doi: 10.1186/s13034-025-00889-2 (PMC11929296; doi:10.1186/s13034-025-00889-2)
Supplement: Supplementary file 1 — Supplementary Material 1 [file 13034_2025_889_MOESM1_ESM.docx]

**Table S1. Baseline characteristics of participants of T1, T2, and T3**

| Variables | T1 (n = 20137)^1^ | T2 (n = 15061) | T3 (n = 14706) | *P^2^* |
| --- | --- | --- | --- | --- |
|  |  |  |  |  |
| **Age, Mean ± SD** | 13.40 ± 1.45 | 13.48 ± 1.47 | 13.44 ± 1.46 | <0.001 |
| **Gender** |  |  |  | <0.001 |
| Boys | 9952 (49.4%) | 7170 (47.6%) | 7341 (49.9%) |  |
| Girls | 10185 (50.6%) | 7891 (52.4%) | 7365 (50.1%) |  |
| **Residence** |  |  |  | <0.001 |
| Country | 7237 (35.9%) | 5242 (34.8%) | 4829 (32.8%) |  |
| Urban | 12900 (64.1%) | 9819 (65.2%) | 9877 (67.2%) |  |
| **Single child** |  |  |  | <0.001 |
| No | 15606 (77.5%) | 11872 (78.8%) | 11692 (79.5%) |  |
| Yes | 4531 (22.5%) | 3189 (21.2%) | 3014 (20.5%) |  |
| **Left-behind child** |  |  |  | 0.068 |
| No | 13383 (66.5%) | 9912 (65.8%) | 9600 (65.3%) |  |
| Yes | 6754 (33.5%) | 5149 (34.2%) | 5106 (34.7%) |  |
| **Father education level** |  |  |  | 0.002 |
| Below high school | 15453 (76.7%) | 11677 (77.5%) | 11525 (78.4%) |  |
| High school or above | 4684 (23.3%) | 3384 (22.5%) | 3181 (21.6%) |  |
| **Mother education level** |  |  |  | <0.001 |
| Below high school | 16032 (79.6%) | 12159 (80.7%) | 11987 (81.5%) |  |
| High school or above | 4105 (20.4%) | 2902 (19.3%) | 2719 (18.5%) |  |
| **Family type** |  |  |  | 0.388 |
| Nuclear family | 15977 (79.3%) | 12024 (79.8%) | 11744 (79.9%) |  |
| Single parent or remarried | 4160 (20.7%) | 3037 (20.2%) | 2962 (20.1%) |  |
| **Alcohol use** |  |  |  | 0.292 |
| Without | 16742 (83.1%) | 12615 (83.8%) | 12280 (83.5%) |  |
| With | 3395 (16.9%) | 2446 (16.2%) | 2426 (16.5%) |  |
| **Smoking use** |  |  |  | 0.756 |
| Without | 18743 (93.1%) | 14049 (93.3%) | 13702 (93.2%) |  |
| With | 1394 (6.9%) | 1012 (6.7%) | 1004 (6.8%) |  |
| **Impaired sleep quality** |  |  |  | 0.153 |
| Without | 14461 (71.8%) | 10825 (71.9%) | 10690 (72.7%) |  |
| With | 5676 (28.2%) | 4236 (28.1%) | 4016 (27.3%) |  |
| **Conduct problems** |  |  |  | 0.154 |
| Without | 18328 (91.0%) | 13796 (91.6%) | 13430 (91.3%) |  |
| With | 1809 (9.0%) | 1265 (8.4%) | 1276 (8.7%) |  |
| **IGD status** |  |  |  | 0.768 |
| Adolescents without problematic gaming | 18155 (90.2%) | 13583 (90.2%) | 13314 (90.5%) |  |
| Problematic gamer | 1982 (9.8%) | 1478 (9.8%) | 1392 (9.5%) |  |
| Gamer with IGD | 516 (2.6%) | 356 (2.4%) | 355 (2.4%) |  |
| **Hyperactivity/inattention problems** |  |  |  | 0.454 |
| Without | 18088 (89.8%) | 13557 (90.0%) | 13296 (90.4%) |  |
| With | 2049 (10.2%) | 1504 (10.0%) | 1410 (9.6%) |  |
| **Emotional problems** |  |  |  | 0.191 |
| Without | 18485 (91.8%) | 13886 (92.2%) | 13535 (92.0%) |  |
| With | 1652 (8.2%) | 1175 (7.8%) | 1171 (8.0%) |  |
| **Peer problems** |  |  |  | 0.376 |
| Without | 18485 (91.8%) | 13886 (92.2%) | 13535 (92.0%) |  |
| With | 1652 (8.2%) | 1175 (7.8%) | 1171 (8.0%) |  |

1Mean ± SD; n (%)

2Welch Two Sample t-test; Pearson's Chi-squared test
